# Supplementary material for: Structural insights into Rad18 targeting by the SLF1 BRCT domains
Source: J Biol Chem. 2023 Sep 23;299(11):105288. doi: 10.1016/j.jbc.2023.105288 (PMC10598736; doi:10.1016/j.jbc.2023.105288)
Supplement: Supplemental information [file mmc1.pdf]

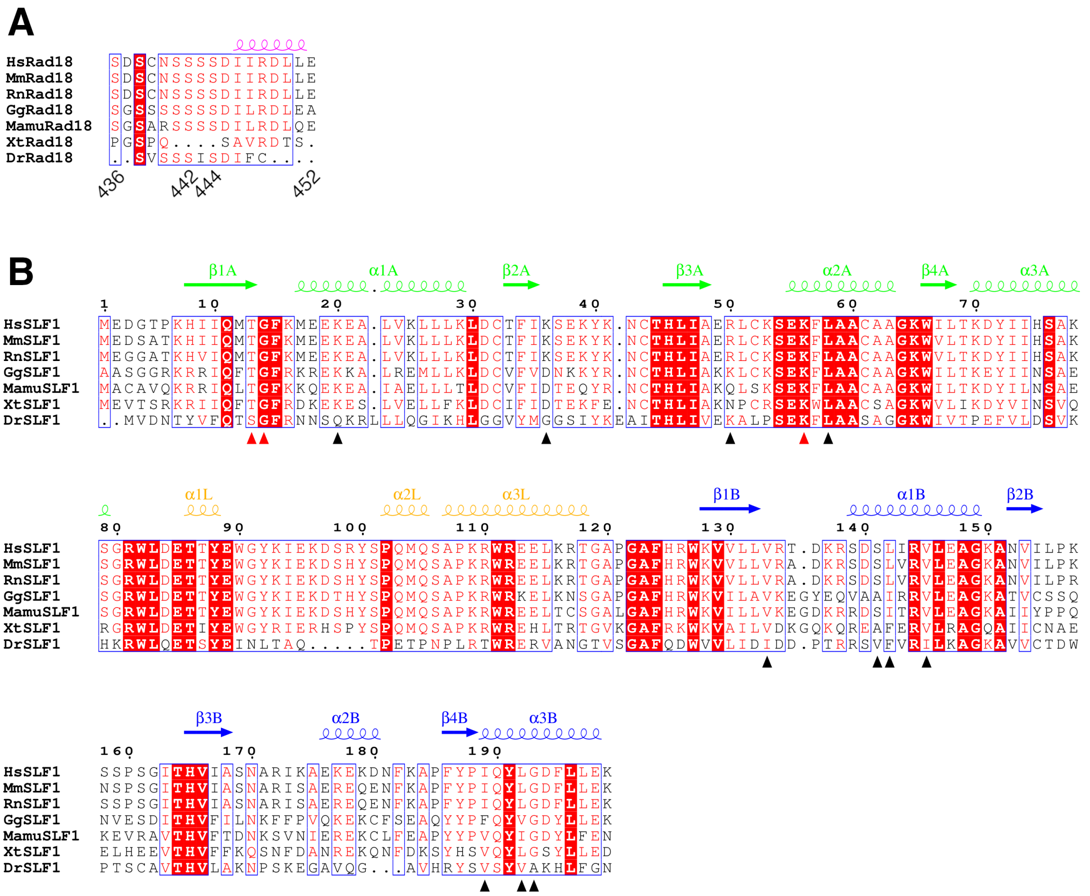

Figure S1 Sequence alignment of Rad18 (A) and SLF1^{tBRCT} (B). Residue numbers and secondary structure elements for the human Rad18 and SLF1^{tBRCT} are shown. The red and black triangles indicate SLF1 residues interacting with pS442 and additional regions in the human Rad18, respectively. Xt, *Xenopus tropicalis* (frog); Dr, *Danio rerio* (zebra fish).

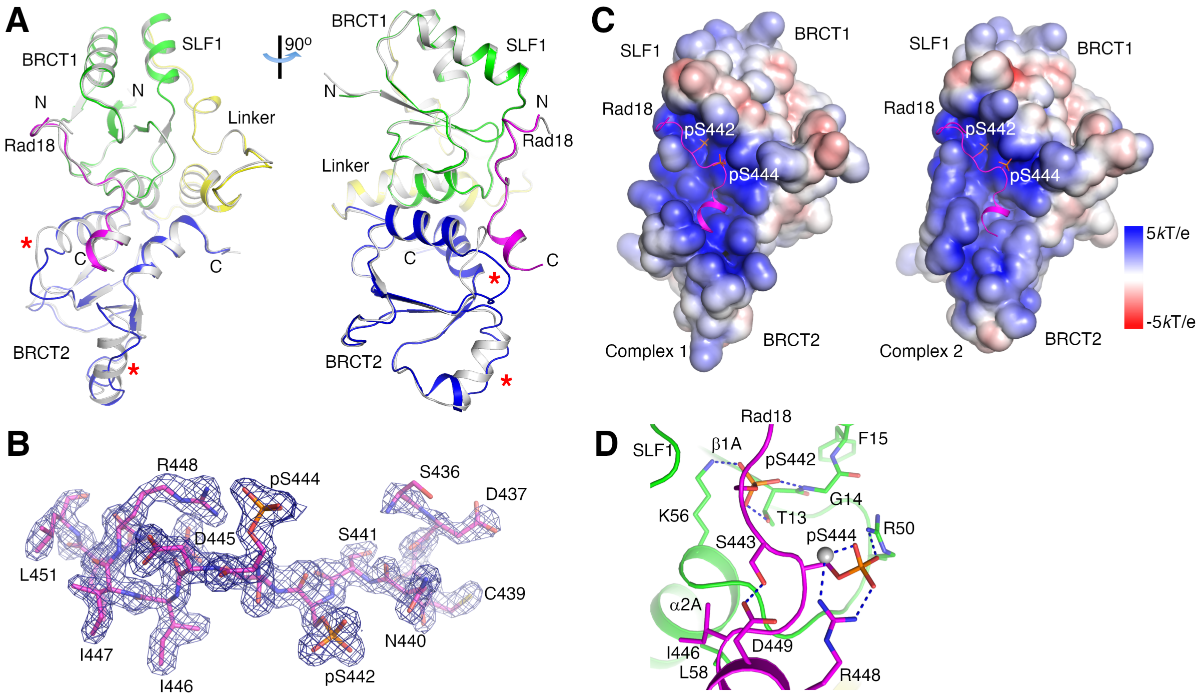

Figure S2 Structure of the SLF1^{tBRCT}-Rad18-2P complex. (A) Structure comparison of the two complexes in the asymmetric unit. The second complex is colored in gray. The red stars indicate regions with large structural differences. (B) Electron density map for the second Rad18-2P peptide in the asymmetric unit. The densities are contoured at 1σ. (C) Surface charge distribution of SLF1^{tBRCT}. Complexes 1 and 2 in the crystal are shown. Cartoon diagrams of the bound Rad18 peptides are shown for reference. (D) Interactions mediated by pS442, pS444 and surrounding regions in Rad18. Structure of complex 2 in the crystal is presented.

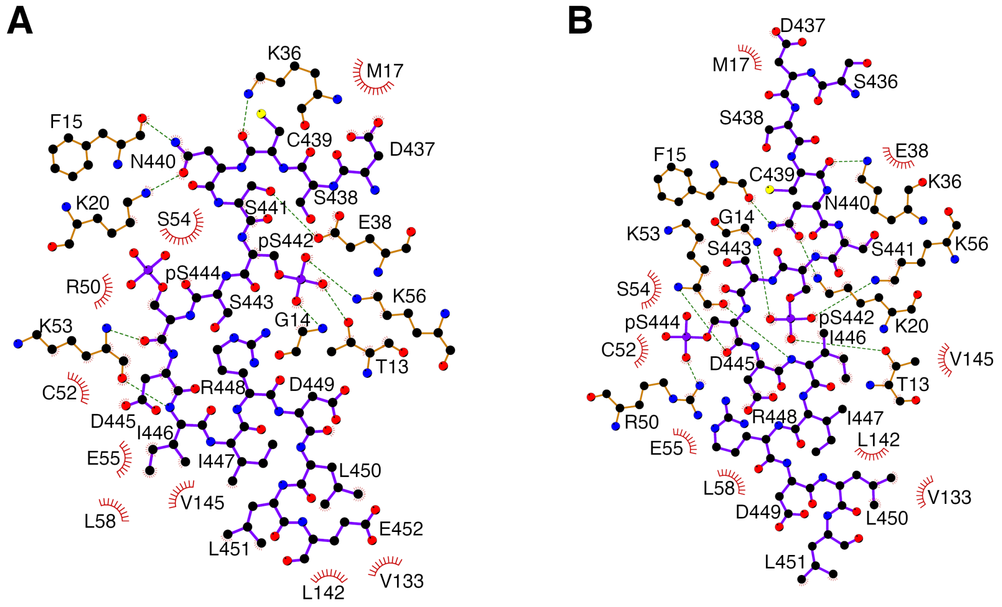

Figure S3 Schematic drawing of the SLF1^{tBRCT}-Rad18-2P interaction. Complexes 1 (A) and 2 (B) in the crystal was presented. The figure is produced with LigPlot (www.ebi.ac.uk/thornton-srv/software/LigPlus/).

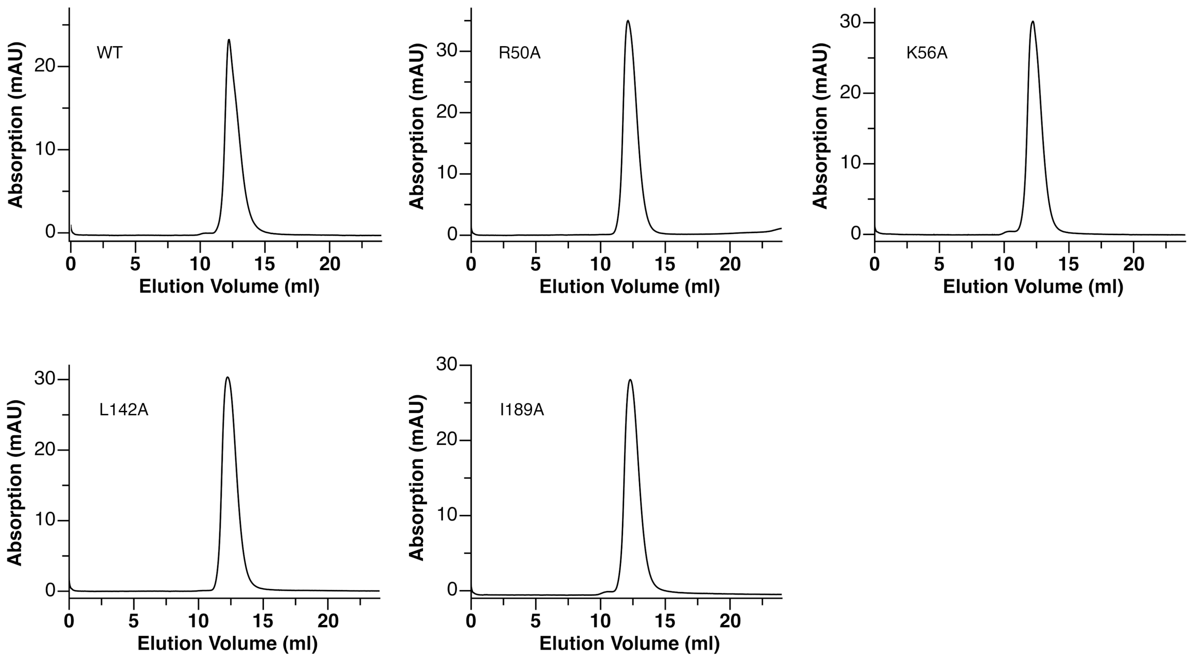

Figure S4 Gel filtration characterization of the wild type (WT) and substituted SLF1^{tBRCT}. The proteins were characterized with a Superdex 75 increase 10/300 column (GE healthcare) with a buffer containing 20 mM Tris (pH 7.5), 200 mM sodium chloride and 2 mM DTT.

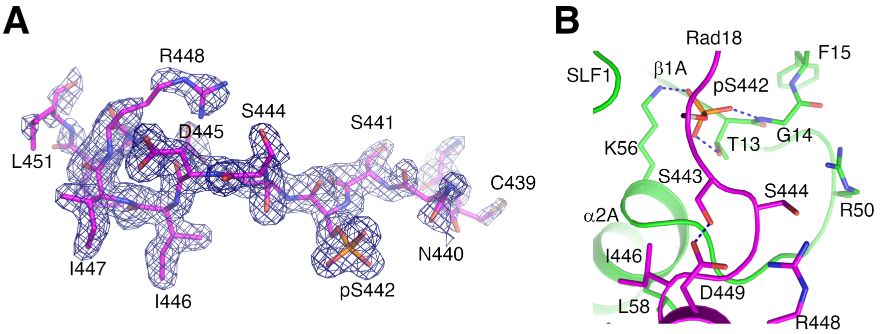

Figure S5 Structure of the SLF1^{tBRCT}-Rad18-pS442 complex. (A) Electron densities for the Rad18-pS442 peptide. Electron densities for the Rad18 peptide bound to SLF1^{tBRCT} molecule 2 in the crystal contoured at 1σ are presented. (B) Interactions mediated by pS442 and surrounding regions in the Rad18-pS442 peptide. Structure of complex 2 in the crystal is presented.

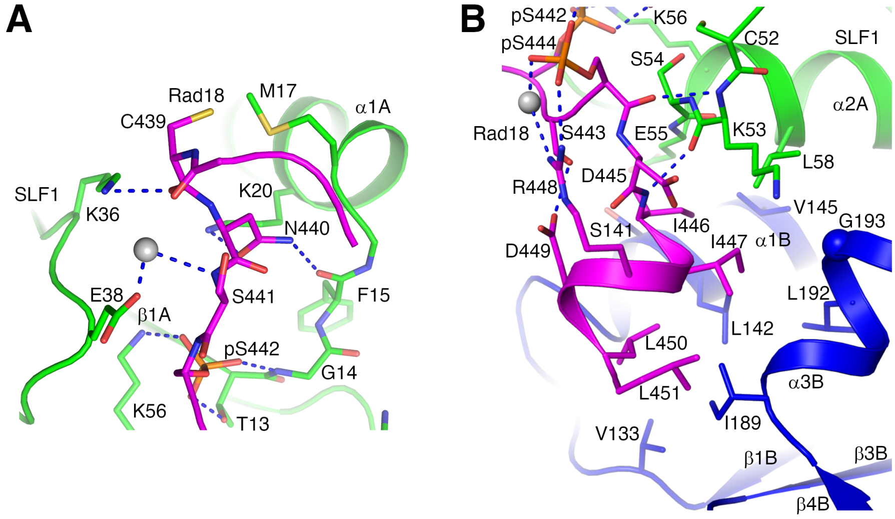

Figure S6 Rad18 regions adjacent to Ser442 and Ser444 contribute to its interaction with $SLF1^{tBRCT}$. Interactions mediated by the N- (A) and C-terminal (B) regions of the Rad18-2P peptide in complex 2 in the crystal are presented.

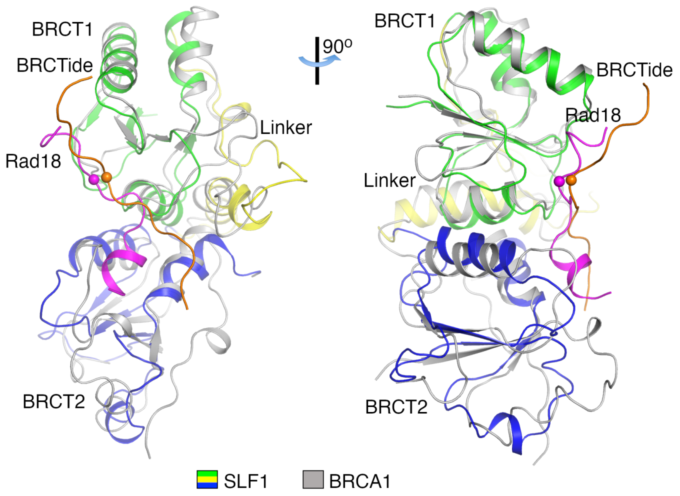

Figure S7 Structural comparison of tBRCTs in SLF1 and BRCA1 (PDB 1T2V, shown in gray). Their structures can be aligned with a RMSD of 1.83 Å for the Cα atoms. The BRCA1-bound BRCTide is shown in brown. The spheres indicate pS442 in Rad18 or the anchoring phosphoserine in BRCTide.

Table S1 Sequences of tBRCT-bound phosphopeptides[1]

PDB	tBRCT factors	Binding partners	Binding partner peptide sequences[2]
	SLF1	Rad18	SDSCNSSSSDIIRDLLE
1T15	BRCA1	BACH1	STSPTFNK
1T29	BRCA1	BACH1	ISRSTSPTFNKQTK
1T2V	BRCA1	BRCTide	GAAYDISQVFPFAKKK
1Y98	BRCA1	CtIP	PTRVSSPVFGAT
3COJ	BRCA1	Acetyl-CoA Carboxylase 1	DSPPQSPTFPEAG
3K0H	BRCA1	Minimal sequence	SPTF-NH_2
3K0K	BRCA1	Minimal sequence	SPTF
3K15	BRCA1-D1840T	Minimal sequence	SPTF-NH_2
3K16	BRCA1-D1840T	Minimal sequence	SPTF
3PXE	BRCA1-E1836K	BACH1	SRSTSPTFNK
4IFI	BRCA1	BAAT	RSPVFS
4IGK	BRCA1	ATRIP	ACSPQFG
4JLU	BRCA1	Abraxas	GFGEYSRSPTF
4U4A	BRCA1	Abraxas	GFGEYSRSPTF
4Y18	BRCA1	Abraxas	GFGEYSRSPTF
4Y2G	BRCA1	Abraxas	YSRSPTF
3L41	BRC1 (BRCT 5/6)	γH2A	KPSQEL
3T7K	Rtt107 (BRCT 5/6)	γH2A	ATKASQEL
3SQD	PTIP (BRCT 5/6)	γH2AX	KKATQASQEY
2VXC	Crb2	γH2A.1	SQEL
5ECG	53BP1	γH2AX	SQEY
2AZM	MDC1	γH2AX	KKATQASQEY
3K05	MDC1-T2067D	Minimal sequence	SQEY-NH_2
3SHV	MCPH1	γH2AX	KKATQASQEY
3SZM	MCPH1	γH2AX	KKATQASQEY
3U3Z	MCPH1	γH2AX	SQEY
3T1N	MCPH1	Cdc27	SDEF
3AL3	TopBP1 (BRCT 7/8)	BACH1	SIYFTPELYD

[1]Sequences of peptides bound to tBRCTs structurally homologous to SLF1[tBRCT] are listed.
[2]Phosphorylated residues are colored in red, disordered residues in the structure are colored in gray. The anchoring phosphoserine or phosphothreonine are aligned.
